# Using stakeholder preferences to select native tree species for reforestation in Lebanon

**DOI:** 10.1007/s11056-018-9648-2

**Published:** 2018-06-01

**Authors:** Arbi J. Sarkissian, Robert M. Brook, Salma N. Talhouk, Neal Hockley

**Affiliations:** 1 School of Environment, Natural Resources and Geography, Bangor University, Bangor, LL57 2UW Wales, UK; 2 Faculty of Agricultural and Food Sciences, American University of Beirut, Riad El Solh, Beirut, 1107-2020 Lebanon; 3 Nature Conservation Center, American University of Beirut, P.O. Box 11-0236, Riad El Solh, Beirut, 1107-2020 Lebanon

**Keywords:** Biodiversity, Forestry, Species selection, Stakeholders, Prioritisation, Reforestation

## Abstract

Using a diverse assemblage of suitable species for reforestation is necessary to enhance biodiversity and ensure resilient forest ecosystems. However, selection of diverse native species for reforestation is difficult, requiring consideration of the preferences of different stakeholders. In this study we identify species to be included in reforestation of an ecologically important watershed in North Lebanon based on ratings produced by stakeholders from Lebanon’s public, private and academic sectors. Twenty-two tree species being produced in Lebanese nurseries were identified as ecologically suitable by experts. Stakeholders (n = 34) were asked to rate these 22 species according to conservation priority and ecological suitability in an online survey. Although there was a high degree of variability in ratings among respondents, those who identified as biodiversity-focused did not differ from those who identified as forestry-focused. Looking within the two foci, we found significant variability among forestry-focused respondents but not among biodiversity-focused respondents. Although there was no significant difference in ratings between biodiversity- and forestry-focussed respondents, the resultant rankings differed considerably. We also found significant variability in preferences within forestry-focussed but not biodiversity-focussed respondents. Weighting by respondents’ knowledge of species had little effect on rankings. The variability in preferences between stakeholders, including the considerable within-group variability we found among forestry-focused respondents, highlights the importance of soliciting preferences from multiple stakeholders when selecting species to be used in reforestation efforts.

**Electronic supplementary material:**

The online version of this article (10.1007/s11056-018-9648-2) contains supplementary material, which is available to authorized users.

## Introduction

Species used in restoration are often chosen to meet short term objectives, such as mitigating on-site degradation (de Baets et al. 2009). Selecting hardier and more adaptable species that are readily available (or easy to produce) can be a more cost-effective means of meeting some of these objectives. But this may also be detrimental to local biodiversity if the species selected are exotic, potentially invasive, or simply a very restricted set of native species (Le Maitre et al. 2011). Species selection can become contentious when there are multiple stakeholders (e.g. experts, policymakers, practitioners and local residents) with potentially divergent aims and objectives in restoration (Pullar and Lamb 2012; Reubens et al. 2011). There may be competing aims between those with professional interests in forestry and those with interests in conserving biodiversity, despite both sectors having some overlapping aims and objectives in reforestation e.g. increasing and/or maintaining forest cover (Angelsen et al. 2012).

The importance of stakeholder participation in facilitating adaptive co-management of complex social-ecological systems has been well documented (Kofinas 2009). Yet stakeholder preferences for species may vary by subjective tastes and the extent of knowledge individuals have about the species and their ecology. Stakeholders involved in restoration or other conservation measures often face difficult trade-offs between social, economic and ecological objectives (Visser et al. 2011). Restoration objectives that support multifunctional landscapes frequently require the assessment of a suite of potential scenarios to minimise future ecosystem service trade-offs (Reed et al. 2013). Eliciting and aggregating heterogeneous preferences is therefore a necessary challenge for engaging multi-stakeholder participation in policy and practice (Newton et al. 2012). Despite recent efforts to address these policy needs in Lebanon, engaging stakeholder participation in forestry and natural resource management issues has been challenging due to  institutional divisions and lack of oversight, compounded by years of sociopolitical instability (Sarkissian et al. 2017).

Lebanon is a relatively small (10,452 km^2^), predominantly mountainous country located in the eastern Mediterranean. It has been recognised as a centre for plant diversity (Davis et al. 1994) harbouring over 2600 vascular plant species with around 12% endemic to the eastern Mediterranean region (Zohary 1973). As a country with ancient roots dating back to the Phoenicians, much of its population over the centuries was concentrated in cities located along a narrow coastline. Settlements at higher elevations gradually increased over time and the resulting larger populations in turn converted forests into croplands and pastures. Forests outside of protected areas continued to decline considerably due to wars, urbanisation, herding, and forest fires (Talhouk et al. 2001).

With threats to plant diversity in the eastern and southern Mediterranean basin increasing, Radford et al. (2011) conducted a rapid assessment of important plant areas (IPAs) in North African and Middle Eastern countries including Lebanon (Yazbek et al. 2010). The team defined a total of twenty IPAs in Lebanon, most located on the western slopes of the Mount Lebanon range with an average elevation of approximately 1200 m (ranging from sea level to 3044 m^1^). Many of Lebanon’s protected areas (e.g. nature reserves, Biosphere reserves, Ramsar sites) occur within these IPAs.

The decline of Lebanon’s forests stimulated efforts to undertake nationwide reforestation and afforestation in the early 1960s, in which *Cedrus libani* Rich. (Cedar of Lebanon) and *Pinus pinea* L. (stone pine) were the main species planted (Regato and Asmar 2011). Since the late 1990s, national stakeholders have committed public and international funds to implement landscape-scale reforestation throughout much of Lebanon including ecologically and culturally important areas (Mohanna et al. 2017). Many stakeholders from public and non-profit sectors have also shown interest in adopting a payments for ecosystem services (PES) approach to fund large-scale forest restoration with a focus on enhancing biodiversity, which requires the planting of a diverse range of species beyond the limited set of predominantly conifer species used in the past (Sarkissian et al. 2017).

It is important to incorporate perceptions of reforestation decision-making from wider society, including preferences for tree species from local stakeholders such as farmers (Garen et al. 2009; Reubens et al. 2011). Prior research in Lebanon revealed that most landowners would prefer to plant forest species with market value, e.g. pine nuts from *Pinus pinea* L., in the absence of payments (Sarkissian et al. 2017). In this study, we aim to complement this research by eliciting the preferences of national reforestation stakeholders (e.g. public sector, NGOs, or private entities) who are potential ‘buyers’ of PES schemes designed to diversify native species used in reforestation.

The objectives of the current study were to (1) identify native tree and shrubby tree species for use in biodiversity-enhancing reforestation in the Bcherre–Ehden IPA in northern Lebanon according to stakeholders in forestry and conservation sectors, (2) to explore the heterogeneity of preferences between and within different stakeholder groups, and (3) examine the implications of weighting by self-reported knowledge when aggregating preferences.

## Methods

A total of 64 native tree and shrub species in Lebanon with growth potentials of > 1 m were identified by experts from the American University of Beirut’s Nature Conservation Center (AUB-NCC). Species were identified using literature on flora in Lebanon and the Levant region (Post 1932; Mouterde 1966) and later updated with the most recent reference on Lebanese flora (Tohmé and Tohmé 2007), noting that nomenclature often differed. The candidate list was then restricted to 22 species that were both (a) available from tree nurseries providing seedlings to reforestation organisations at the time this study was being conducted and (b) considered ecologically suitable for the site (altitude range of 1000–1500 m; severe summer dry season) by experts at the AUB-NCC. This site is characteristic of eu-mediterranean (> 1000 m) to oro-mediterranean (> 2000 m) bioclimatic zones. Average annual precipitation in this region ranges from 850 to 950 mm, mainly from October to May, with the heavier rains and snowfall occurring between December and March (Jomaa 2008). The vegetation types are typical of Mediterranean forest, woodland and scrub communities containing coniferous, deciduous and mixed forest/woodlands (Abi-Saleh and Safi 1988).

An online survey was conducted with stakeholders in the forestry and conservation sectors in May, 2013 (Online Resource 1). The survey (in both English and Arabic) was delivered via personalised email invitations with a brief description of the study and a link to the online survey. Respondents were first asked to rate each of the 22 species listed as either: high, medium, or low conservation priorities for inclusion in reforestation in the research site (which was described in the email). They also had the option to select ‘Ecologically unsuitable’ if they believed the species should not be planted in the site described, or ‘Don’t know this species’ if they had insufficient knowledge. A hyperlink was provided next to each species directing the respondents to the ‘Euro + Med PlantBase’ website (2011) providing extra details of the species, e.g. authorities, nomenclature, synonyms, references, distribution. An optional second question asked participants to list up to five additional species that were both suitable for this research site and which they would consider as high conservation priorities for reforestation. The third and fourth questions asked respondents to describe their profession (‘Academia’, ‘Government/Public sector’, ‘Private sector’, and ‘Other’) and sectoral focus (‘Biodiversity conservation’, ‘Forestry’, ‘Agriculture’, and ‘Other’).

Stakeholders selected to participate in this online survey were identified with the help of colleagues from AUB-NCC who provided us with contacts from their professional networks. Contact details and additional candidates were also obtained through searching the public domain and through snowballing. The online survey was created and administered using LimeSurvey^®^. Statistical analyses were conducted using SPSS^®^ (version 22). The study received ethics approval from the Institutional Review Board (IRB) of the American University of Beirut (FAFS.ST.07).

**Fig. 1:**
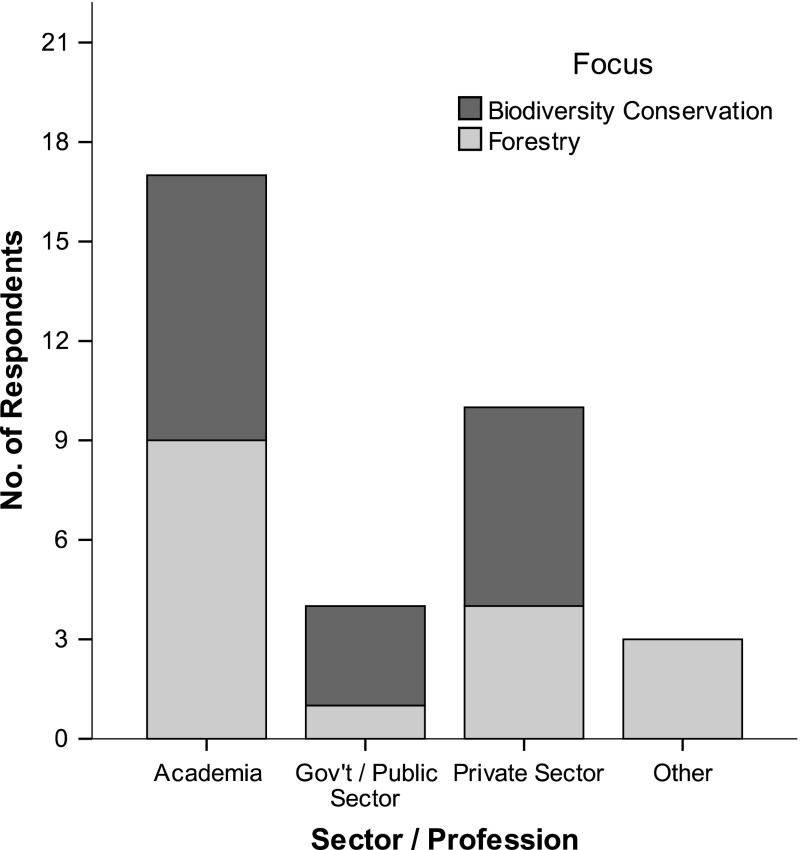
Respondents classified according to sector/profession and focus. Participants who indicated ‘Other’ under *Sector*/*Profession* indicated forestry expert, international agencies, and researcher as open responses (created on SPSS v.20)

In total, 80 individual invitations (with LimeSurvey^®^ tokens) were sent to respondents from various academic and professional backgrounds. Thirty-four respondents (44%) fully completed the survey from early May to mid-June, 2013. The sample included respondents from academic institutions (n = 17), private sector/NGOs (n = 10), public institutions (n = 4) and other/unspecified (n = 3), with professional foci split between forestry and biodiversity conservation (Fig. 1).

We used four different ways to aggregate rankings and compared the results. In all instances, ordinal ratings of importance (low, medium, and high) were given numerical values (1–3, respectively) and “ecologically unsuitable” coded as 0. In the first instance, we produced aggregate rankings based on each species’ mean score (0–3) treating ‘Don’t know this species’ as missing values (i.e. unweighted). We then produced two rankings split between biodiversity conservation focussed and forestry focussed individuals (n = 17 for each). To compare preferences across stakeholder groups we used the Mann–Whitney *U* test to identify if there were any significant differences between the two foci (biodiversity conservation and forestry), and Friedman’s ANOVA to explore within foci variation. Lastly, we produced ranks weighting for how knowledgeable respondents were by treating missing values as an index of knowledge based on the proportion of total species (n = 22) they knew. The standard error of the mean was used to rank means that were tied (i.e. those with lower standard errors were ranked higher).

## Results

**Fig. 2:**
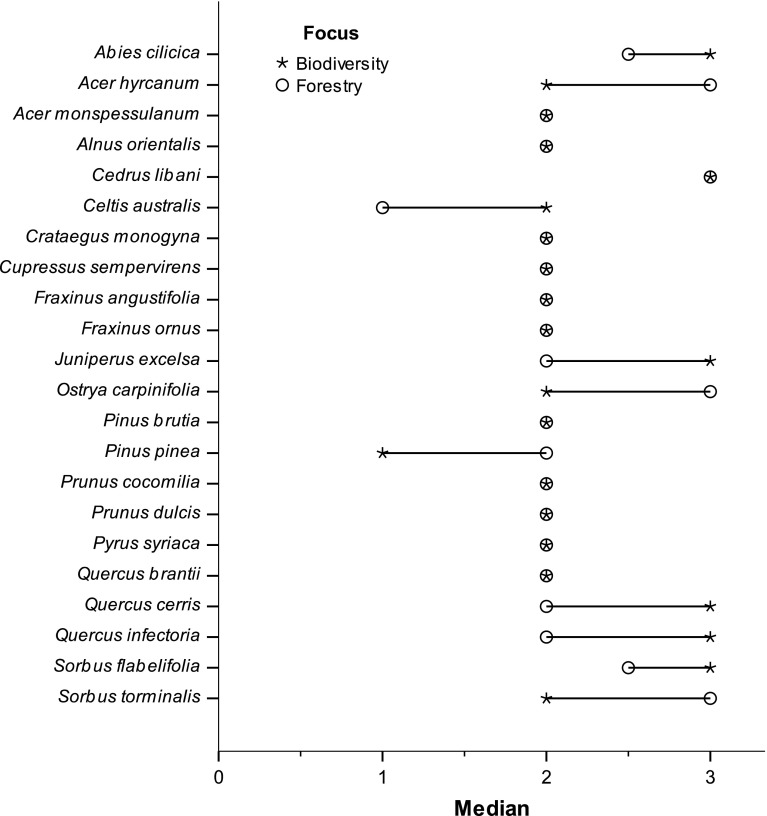
Median ratings of species split between two foci—Biodiversity Conservation and Forestry (created on SPSS v.20). 0 = ecologically unsuitable, 1 = low conservation priority, 2 = medium conservation priority, and 3 = high conservation priority

Cedar of Lebanon (*Cedrus libani* Rich.) received the largest number of ‘High’ ratings by respondents. Grecian juniper (*Juniperus excelsa* M. Bieb.) received the most ratings for ecologically unsuitable (n = 5). Only six of the 22 species were rated (i.e. were known) by all 34 respondents. Median ratings were equal for respondents in both groups for 12 of the 22 species (Fig. 2). Eight of the 22 species were unfamiliar to six or more respondents.

There was significant variation in ratings across the 23 respondents familiar with the remaining 14 species [Friedman’s ANOVA, χ^2^ (13) = 62.25, *p *< 0.001]. Friedman’s tests on each subset of respondents indicated no significant variation between respondents within the biodiversity focus [Friedman’s ANOVA, χ^2^ (5) = 31.47, *p *> 0.05] but significant variation was found within the forestry focus [Friedman’s ANOVA, χ^2^ (9) = 50.55, *p *< 0.01]. Post-hoc Mann–Whitney *U* tests showed no significant difference (*p *> 0.05) in ratings between forestry- and biodiversity-focused respondents for the 22 species, even before correction for multiple comparisons.

The rankings produced (based on mean scores) from these two groups’ ratings did, however, appear noticeably different (Table 1). For example, *Abies cilicica* (Ant. & Kotschy) Carriére (Cilician fir) was top-ranked by biodiversity-focussed respondents and eleventh by the forestry-focussed ones. Conversely, *Acer hyrcanum* subsp. *tauricola* (Boiss. & Balansa) Yalt. (Taurus maple) and *Fraxinus ornus* L. (manna ash) were ranked second and third by forestry-focussed respondents, but 14th and 12th (respectively) by biodiversity-focussed ones. Both groups had fairly similar averages for knowledge of species (aggregated), although differences varied from species to species. Finally, weighting respondents by the proportion of species of which they claimed knowledge made little difference to rankings.

**Table 1:** Rankings of species produced from biodiversity-focused (left) and forestry-focused (right) respondents. The rankings present both stakeholder knowledge of (i.e. lower of “% Known” was based on frequency of those who selected “Don’t know this species”) and preference for inclusion of 22 suitable species in the designated Important Plant Area (IPA) in Bcherre-Ehden, Lebanon

Rank	Biodiversity-focussed				Rank	Forestry-focussed			
	Species	Mean	S.E. of mean	% Known		Species	Mean	S.E. of mean	% Known
1	*Abies cilicica*	2.588	0.193	100	1	*Cedrus libani*	2.647	0.170	100
2	*Cedrus libani*	2.588	0.211	100	2	*Acer hyrcanum*	2.467	0.192	87
3	*Quercus infectoria*	2.529	0.125	100	3	*Fraxinus ornus*	2.400	0.163	87
4	*Quercus cerris*	2.529	0.151	100	4	*Sorbus torminalis*	2.385	0.213	69
5	*Juniperus excelsa*	2.438	0.302	94	5	*Ostrya carpinifolia*	2.308	0.237	69
6	*Sorbus flabelifolia*	2.400	0.190	87	6	*Pyrus syriaca*	2.294	0.166	100
7	*Sorbus torminalis*	2.333	0.187	87	7	*Sorbus flabelifolia*	2.250	0.250	58
8	*Crataegus monogyna*	2.294	0.143	100	8	*Crataegus monogyna*	2.235	0.161	100
9	*Pyrus syriaca*	2.250	0.144	94	9	*Quercus infectoria*	2.235	0.182	100
10	*Quercus brantii*	2.231	0.201	69	10	*Quercus cerris*	2.235	0.202	100
11	*Prunus cocomilia*	2.154	0.154	69	11	*Abies cilicica*	2.188	0.262	94
12	*Fraxinus ornus*	2.083	0.260	58	12	*Cupressus sempervirens*	2.176	0.214	100
13	*Acer monspessulanum*	2.071	0.195	79	13	*Acer monspessulanum*	2.071	0.245	79
14	*Acer hyrcanum*	2.059	0.234	100	14	*Prunus dulcis*	2.000	0.195	87
15	*Cupressus sempervirens*	1.938	0.193	94	15	*Juniperus excelsa*	2.000	0.195	87
16	*Ostrya carpinifolia*	1.846	0.274	69	16	*Prunus cocomilia*	1.933	0.153	87
17	*Pinus brutia*	1.765	0.265	100	17	*Pinus brutia*	1.882	0.225	100
18	*Prunus dulcis*	1.750	0.194	94	18	*Fraxinus angustifolia*	1.786	0.281	79
19	*Alnus orientalis*	1.692	0.263	69	19	*Pinus pinea*	1.765	0.265	100
20	*Fraxinus angustifolia*	1.667	0.225	58	20	*Quercus brantii*	1.471	0.259	100
21	*Celtis australis*	1.563	0.203	94	21	*Alnus orientalis*	1.467	0.215	87
22	*Pinus pinea*	1.529	0.194	100	22	*Celtis australis*	1.267	0.206	87
	Average	2.104		87			2.066		89

0 = ecologically unsuitable, 1 = low conservation priority, 2 = medium conservation priority, and 3 = high conservation priority

## Discussion

Previous studies have found contrasting perspectives between forestry and biodiversity conservation, especially with regards to international-scale policies such as Reducing Emissions from Deforestation and forest Degradation (REDD+) (Angelsen et al. 2012). While we found a high degree of variability in rating among respondents, which was not fully explained by professional focus (forestry versus biodiversity conservation), there was no clear pattern of ratings between these two groups. Similar studies examining stakeholder preferences for tree species found much clearer differences between men and women, where roles and access to resources also differed based on gender (Ureta et al. 2016). In contrast, the groups in our study were determined based on respondents choosing whether the main focus of their profession was biodiversity conservation or forestry. Investigating preferences for forests scenes, Kearney and Bradley (2011) found that group membership failed to account for differences in preferences since presumably distinct groups (e.g. natural resource agencies) often comprise individuals with a wide-range of expertise and professional backgrounds.

Even where there seem to be no consistent differences in preferences between groups of stakeholders, disagreements appear to exist amongst stakeholders within those groups. This could be because these categories failed to adequately represent respondents’ perspectives. However, most respondents (91%) readily self-identified with one or another of these categories. Instead it seems that preferences are diverse even within these groupings, something which has implications for planning conservation-focussed reforestation: it implies that even within a specific group, a sufficiently broad sample of stakeholders should be consulted, rather than relying on a few representatives. Another important aspect is to consider how attitudes and knowledge factor into preferences, in particular how respondents interepret their own preferences (Kearney and Bradley 2011).

Interestingly, while most forestry-focussed respondents rated *Sorbus torminalis* (L.) Crantz. and *Ostrya carpinifolia* Scop. high (ranked fourth and fifth, respectively), nearly a third did not know the species. And *Sorbus flabelifolia* (Spach) Hedl. was ranked seventh despite nearly half of the forestry-focussed respondents having no knowledge of this species. In contrast, most biodiversity-focussed respondents were familiar with the top nine ranked species. Similarly a substantial number of missing values (i.e. ‘Don’t know this species’) from both foci shows the value of consulting a broad sample of respondents, since few respondents will be familiar with all candidate species. It also underlines the need for standardising and updating the ever-changing taxonomic nomenclature, thus an opportunity for stakeholders to work collectively towards improving plant identification and knowledge dissemination more effectively.

Our survey did not explore the criteria respondents were using to judge conservation importance, e.g. rarity of those species in the wild, or their importance in underpinning ecosystem functioning or in supplying particular ecosystem services. For example, Kermes oak (*Quercus coccifera* L.)^2^ is a common species found in much of the eastern Mediterranean basin (Ganatsas et al. 2012), yet was listed by six respondents (three from each foci) as an additional species considered to be both suitable and of high conservation priority for the site (Online Resource 2), suggesting that respondents were not predominantly considering rare species.

Reforestation stakeholders have expressed interests in developing asset-building PES schemes aimed at enhancing Lebanon’s biodiversity by paying landowners to plant diverse native species (Sarkissian et al. 2017). We expected the respondents to be knowledgeable about most of the common native tree species in Lebanon, and preferences of experts would therefore play an invaluable role in the design and implementation of future PES schemes. Identifying species of high conservation value fulfils the primary objective of enhancing biodiversity along with other on- and off-site ecosystem services. However, we acknowledge that it should not be the only criterion for species selection. In Mediterranean ecosystems, silvicultural treatments using pioneer species (e.g. *Pinus* spp.) are highly effective at restoring degraded landscapes (Ganatsas et al. 2012), which can later be supplemented with rarer species once those habitats develop. Interestingly, respondents in both groups rated the two pine species (*Pinus pinea* L. and *P. brutia* Ten.) as of relatively low conservation priority.

It is also important to acknowledge that preferences are inherently subjective and often heterogenous. A recent study on stakeholder perceptions of a portfolio of tree species to be considered for plantation forestry in New Zealand described how assumptions about various attributes of those species were inaccurate (Smaill et al. 2014). That study also found similar preference heterogeneity to our own study, and showed how stakeholders may hold biases towards more familiar species, which perhaps was reflected in our respondents’ ratings as well. Such biases are not uncommon in conservation planning (Coppolillo et al. 2004). However it is interesting to note that weighting responses by respondents’ level of knowledge did not greatly affect the rankings. Acknowledging biases in the evaluation of studies such as these are therefore critical, especially since the expertise of individuals within or across different professional backgrounds may not only vary, but also be lacking (Smaill et al. 2014; Kearney and Bradley 2011).

Lastly, reforestation stakeholders will need to consider farmers as important stakeholders in their efforts to plant forest trees on private lands. Understanding farmer perceptions of reforestation (or afforestation) and eliciting their preferences for species are important research considerations. Our previous study showed that most Lebanese farmers strongly preferred productive species over non-productive native species, and that paying landowners to plant species of little or no private benefits could be costly (Sarkissian et al. 2017). It is important to consider these trade-offs to identify cost-effective strategies, which will likely require providing a mixture of productive species of low conservation priority (e.g. *Pinus pinea* L.) to be planted along with those of high-conservation-value.

Multi-stakeholder engagement efforts are challenging yet vital for maintaining resilient forest and woodland ecosystems, biodiversity conservation, and human well-being. Establishing new forests to meet multiple objectives (e.g. social, economic and ecological) often involves difficult trade-offs (Carnus et al. 2006). A better understanding of stakeholder typologies is needed to facilitate collaborative engagement between stakeholders from different research and policy fields (or foci), including social scientists and local stakeholders involved in community-based projects (Reed et al. 2009). Our study therefore highlights the need for promoting multi-stakeholder engagement towards policy efforts aimed at restoring and enhancing ecosystem services in Lebanon. Our results show that it is difficult to clearly ascertain stakeholder preferences without consulting multiple stakeholder groups and multiple individuals within a particular stakeholder group. We recommend that future studies further examine stakeholder preferences using qualitative methods, particularly since the population of knowledgeable stakeholders may often be quite small. These efforts would help both researchers and stakeholders to better understand where shared and diverging perspectives exist that could be used to inform quantitative surveys such as the one reported in this paper.

## Electronic supplementary material

Below is the link to the electronic supplementary material.

Supplementary material 1 (PDF 618 kb)

Supplementary material 2 (DOCX 19 kb)

## Footnotes

1 Qornet es-Saouda (highest peak in Lebanon).

2 Syn: *Quercus calliprinos* Webb.

## References

[CR1] Abi-Saleh B, Safi S (1988). Carte de la vegetation du Liban. Ecol Mediter.

[CR2] Angelsen A, Brockhaus M, Sunderlin WD, Verchot LV (2012). Analysing REDD: challenges and choices.

[CR3] Carnus J, Parrotta JA, Brockerhoff EG, Arbez M, Jactel H, Kremer A, Lamb D, O’Hara K, Walters BB (2006). Planted forests and biodiversity. J For.

[CR4] Coppolillo P, Gomez H, Maisels F, Wallace R (2004). Selection criteria for suites of landscape species as a basis for site-based conservation. Biol Conserv.

[CR5] Davis SD, Heywood VH, Hamilton AC, Heywood VH, Davis SD (1994). Europe, Africa, South West Asia and the Middle East. Centres of plant diversity: a guide and strategy for their conservation.

[CR6] de Baets S, Poesen J, Reubens B, Muys B, de Baerdemaeker J, Meersmans J (2009). Methodological framework to select plant species for controlling rill and gully erosion: application to a Mediterranean ecosystem. Earth Surf Process Landf.

[CR7] Euro + Med Plantbase (2011) The information resource for Euro-Mediterranean plant diversity. http://ww2.bgbm.org/EuroPlusMed/query.asp. Accessed 15 Sept 2013

[CR8] Ganatsas P, Tsitsoni T, Tsakaldimi M, Zagas T (2012). Reforestation of degraded Kermes oak shrublands with planted pines: effects on vegetation cover, species diversity and community structure. New Forest.

[CR9] Garen EJ, Saltonstall K, Slusser J, Mathias S, Ashton MS, Hall JS (2009). An evaluation of farmers’ experiences planting native trees in rural Panama: implications for reforestation with native species in agricultural landscapes. Agrofor Syst.

[CR10] Jomaa I (2008) Analyse diachronique de la fragmentation des forêts du Liban. Doctoral thesis. Université Paul Sabatier-Toulouse III

[CR11] Kearney AR, Bradley GA (2011). The Effects of viewer attributes on preference for forest scenes: contributions of attitudes, knowledge, demographic factors, and stakeholder group membership. Environ Behav.

[CR12] Kofinas GP, Chapin FS, Kofinas GP, Folke C (2009). Principles of ecosystem stewardship: resilience-based natural resource management in a changing world. Adaptive co-management in social-ecological governance.

[CR13] Le Maitre DC, Gaertner M, Marchante E, Ens E, Holmes PM, Pauchard A (2011). Impacts of invasive Australian acacias: implications for management and restoration. Divers Distrib.

[CR14] Mohanna C, Adada F, Besacier C (2017) Forest and landscape restoration in Lebanon. http://www.fao.org/in-action/forest-landscape-restoration-mechanism/resources/detail/en/c/412643/. Accessed 17 Dec 2016

[CR15] Mouterde PSJ (1966). Nouvelle flore du Liban et de la Syrie.

[CR16] Newton AC, Hodder K, Cantarello E, Perrella L, Birch JC, Robins J, Douglas S, Moody C, Cordingley J (2012). Cost-benefit analysis of ecological networks assessed through spatial analysis of ecosystem services. J Appl Ecol.

[CR17] Post GE (1932). Flora of Syria, Palestine and Sinai.

[CR18] Pullar D, Lamb D, Stanturf J, Madsen P, Lamb D (2012). A tool for comparing alternative forest landscape restoration scenarios. A goal-oriented approach to forest landscape restoration.

[CR19] Radford EA, Catullo G, de Montmollin B (2011). Important plant areas of the south and east Mediterranean region: priority sites for conservation.

[CR20] Reed MS, Graves A, Dandy N, Posthumus H, Hubacek K, Morris J, Prell C, Quinn CH, Stringer LC (2009). Who’s in and why? A typology of stakeholder analysis methods for natural resource management. J Environ Manage.

[CR21] Reed MS, Hubacek K, Bonn A, Burt TP, Holden J, Stringer LC (2013). Anticipating and managing future trade-offs and complementarities between ecosystem services. Ecol Soc.

[CR22] Regato P, Asmar F (2011). Analysis and evaluation of forestation efforts in Lebanon.

[CR23] Reubens B, Moeremans C, Poesen J, Nyssen J, Tewoldeberhan S, Franzel S, Deckers J, Orwa C, Muys B (2011). Tree species selection for land rehabilitation in Ethiopia: from fragmented knowledge to an integrated multi-criteria decision approach. Agrofor Syst.

[CR24] Sarkissian AJ, Brook RM, Talhouk SN, Hockley N (2017). Asset-building payments for ecosystem services: assessing landowner perceptions of reforestation incentives in Lebanon. For Syst.

[CR25] Smaill SJ, Bayne KM, Coker GWR, Paul TSH, Clinton PW (2014). The right tree for the job? Perceptions of species suitability for the provision of ecosystem services. Environ Manage.

[CR26] Talhouk SN, Zurayk R, Khuri S (2001). Conservation of the coniferous forests of Lebanon: past, present and future prospects. Oryx.

[CR27] Tohmé G, Tohmé HS (2007). Illustrated flora of Lebanon.

[CR28] Ureta JU, Evangelista KPA, Habito CMD, Lasco RD (2016). Exploring gender preferences in farming system and tree species selection: perspectives of smallholder farmers in Southern Philippines. J Environ Sci Manag.

[CR29] Visser M, Maughan N, Ouled Belgacem A, Neffati M (2011). Stakeholder views on restoring depleted cereal fallows in arid Tunisia: societal barriers and possible crevices. J Arid Environ.

[CR30] Yazbek MM, Houri N, El-Zein M, Safi S, Sinno-Seoud N, Talhouk SN (2010). Important plant areas in Lebanon: a preliminary study based on published literature and consultations with national experts.

[CR31] Zohary M (1973). Geobotanical foundations of the Middle East.

