# Supplementary material for: Using stakeholder preferences to select native tree species for reforestation in Lebanon
Source: New For (Dordr). 2018 Jun 1;49(5):637–47. doi: 10.1007/s11056-018-9648-2 (PMC6096903; doi:10.1007/s11056-018-9648-2)
Supplement: Supplementary file 1 — Supplementary material 1 (PDF 618 kb) [file 11056_2018_9648_MOESM1_ESM.pdf]

Conservation priorities for reforestation in Lebanon - أولويات المحافظة في عملية إعادة التحريج في لبنان

0% 100%

: *

Please rate each of the following species as high, medium, or low conservation priorities for inclusion in reforestation of this area (eastern part of Zghorta, Bcharre and Batroun districts). If you consider this species as being unsuitable for this area, despite its overall importance, please tick 'Ecologically Unsuitable'.

الرجاء تقييم الأصناف التالية حسب أولوية استخدامها في عملية إعادة التحريج في منطقة شرق قضائي بشرّي والبترون (أولوية عالية - high, أولوية متوسطة - medium, أولوية منخفضة - low).

- يرجى وضع إشارة على "ecologically unsuitable" في حال اعتبارك هذه الأصناف غير مناسبة إيكولوجيًا للمكان بالرغم من أهميتها.
- يرجى وضع إشارة على "don't know this species"، في حال عدم معرفتك بالصنف.

	High	Med	Low	Eco-logically Un-suitable	Don't know this species
Pinus brutia Ten. - Calabrian pine - صنوبر تركي [more info]					
Fraxinus ornus L. - Manna ash - الدردار المزهر [more info]					
Cupressus sempervirens L. - Mediterranean cypress - السرو العمودي [more info]					
Pyrus syriaca Boiss. - Syrian pear - إجاص سوري [more info]					
Quercus cerris L. [syn: Q. pseudocerris Boiss.] - Turkey oak - العزرأو البلوط الشعري [more info]					
Acer monspessulanum subsp microphyllum (Boiss.) Bornm. - Montpellier maple - قيقب كوردي [more info]					
Ostrya carpinifolia Scop. - Hop-hornbeam - مرّان [more info]					
Abies cilicica (Antoine & Kotschy) Carrière - Cilician fir - شوح [more info]					
Sorbus flabellifolia (Spach.) Schneider - Fan-leaved service tree - غبيرة مروحية الورق [more info]					
Juniperus excelsa M. Bieb. - Greek juniper - لزّاب [more info]					
Acer hyrcanum subsp. tauricola (Boiss. & Balansa) Yalt. - Taurus maple - قيقب طروسي [more info]					
Prunus cocomilia Ten. - Bear plum - برقوق [more info]					

Quercus infectoria Olivier - Cyprus or Aleppo oak - العفص أو الملول [more info]	○	○	○	○	○
Crataegus monogyna Jacq. – Single-seeded hawthorn - زعرور أحمر [more info]	○	○	○	○	○
Sorbus torminalis (L.) Crantz. - Wild service tree - غبيرة [more info]	○	○	○	○	○
Prunus dulcis (Mill.) D.A. Webb. - Common almond - لوز [more info]	○	○	○	○	○
Fraxinus angustifolia subsp. syriaca (Boiss.) Yalt. - Syrian ash - الدردار القوقازي [more info]	○	○	○	○	○
Alnus orientalis Decaisne - Oriental alder - نغت [more info]	○	○	○	○	○
Pinus pinea L. - Stone pine - صنوبر مثمر [more info]	○	○	○	○	○
Quercus brantii subsp. look (Kotschy) Mouterde - Mt. Tabor oak - بلوط إيراني [more info]	○	○	○	○	○
Celtis australis L. - Mediterranean hackberry - ميس [more info]	○	○	○	○	○
Cedrus libani Rich. - Cedar of Lebanon - أرز لبنان [more info]	○	○	○	○	○

Please note that when using the hyperlink ('more info') you will need to agree to the licence term first, then click on the link again to access the web page

:
Please indicate if there are any other species you would consider to be suitable for this area AND a high conservation priority for reforestation

الرجاء إضافة أصناف غير موجودة أعلاه، تعتبرها المحافظة عليها أولوية

1 ______
2 ______
3 ______
4 ______
5 ______

Please list up to five species only using common English or Arabic names and include scientific name if known
الرجاء ذكر أسماء خمسة أنواع على الأكثر؛ يمكنكم إستخدام الاسم العلمي أو الاسم الشائع بالعربية أو الانكليزية

: *
Please indicate which category best describes your occupation (whether active or retired)

الرجاء اختيار المجال الأقرب من وظيفتك

Choose one of the following answers

- Academia: scientist, researcher, lecturer (University and/or research center) - (أكاديميا (عالم، باحث، محاضر في الجامعة أو مركز أبحاث، إلخ
- Government / public sector (incl. local/municipal): policy, management and planning - حكومي/قطاع عام (سياسي، إداري، منظم)
- Private sector / 3rd sector - NGO, consultancy, business - قطاع خاص (جمعية غير حكومية، استشاري، تجاري
- Other - أُخرى

: *
How would you best describe your professional focus?

يرجى وضع علامة على المحور الأقرب لمجال اختصاصك

Choose one of the following answers

- Biodiversity conservation - محافظة على التنوّع البيولوجي
- Forestry - تحريج / غابات
- Agriculture - الزراعة
- Other - أُخرى

Resume later | << Previous | Submit | Exit and clear survey

This survey is currently not active. You will not be able to save your responses.
