# Supplementary material for: Using stakeholder preferences to select native tree species for reforestation in Lebanon
Source: New For (Dordr). 2018 Jun 1;49(5):637–47. doi: 10.1007/s11056-018-9648-2 (PMC6096903; doi:10.1007/s11056-018-9648-2)
Supplement: Supplementary file 2 — Supplementary material 2 (DOCX 19 kb) [file 11056_2018_9648_MOESM2_ESM.docx]

**Online Resource 2**

Title: Using stakeholder preferences to select native tree species for reforestation in Lebanon

Journal: *New Forests*

Authors & Affiliations: Arbi J. Sarkissian^1*^, Rob M. Brook^1^, Salma N. Talhouk^2^, and Neal Hockley^1^

*^1^School of Environment, Natural Resources and Geography, Bangor University, Bangor, Wales, LL57 2UW, United Kingdom*

*^2^Faculty of Agricultural and Food Sciences, American University of Beirut, 1107-2020, Lebanon*

*Email: arbi.sarkissian@outlook.com

Table 3: Additional native species mentioned by 18 respondents showing frequency mentioned and availability in the market

|  |  | Frequency | | | |  | |
| --- | --- | --- | --- | --- | --- | --- | --- |
| Species | Family | Biodiversity (n=8) | Forestry (n=10) | Total | Available^†^ | |  |
| *Quercus coccifera* ^a^ | Fagaceae | 3 | 3 | 6 | √ | |  |
| *Crataegus azarolus* | Rosaceae | 2 | 3 | 5 |  | |  |
| *Malus trilobata* | Rosaceae | 1 | 4 | 5 | √ | |  |
| *Styrax officinalis* | Styracaceae | 1 | 4 | 5 | √ | |  |
| *Juniperus oxycedrus* | Cupressaceae | 2 | 2 | 4 |  | |  |
| *Juniperus drupacea* | Cupressaceae | 1 | 2 | 3 |  | |  |
| *Myrtus communis* | Myrtaceae |  | 2 | 2 | √ | |  |
| *Pistacia palaestina* | Anacardiaceae | 1 | 1 | 2 | √ | |  |
| *Prunus argentea* ^b^ | Rosaceae | 1 | 1 | 2 |  | |  |
| *Quercus cedrorum* | Fagaceae | 1 | 1 | 2 |  | |  |
| *Acer hermoneum* | Aceraceae |  | 1 | 1 | √ | |  |
| *Arbutus andrachne* | Ericaceae |  | 1 | 1 | √ | |  |
| *Ceratonia siliqua* | Fabaceae |  | 1 | 1 | √ | |  |
| *Cornus sanguinea* ssp*. australis* | Cornaceae |  | 1 | 1 |  | |  |
| *Cydonia* spp. (Quince) | Rosaceae |  | 1 | 1 |  | |  |
| *Juniperus foetidissima* | Cupressaceae |  | 1 | 1 |  | |  |
| *Laurus nobilis* | Lauraceae |  | 1 | 1 | √ | |  |
| *Pistacia atlantica* | Anacardiaceae | 1 |  | 1 |  | |  |
| *Pistacia lentiscus* | Anacardiaceae | 1 |  | 1 | √ | |  |
| *Platanus orientalis* | Platanaceae |  | 1 | 1 | √ | |  |
| *Prunus arabica* ^c^ | Rosaceae | 1 |  | 1 |  | |  |
| *Prunus mahaleb* | Rosaceae |  | 1 | 1 |  | |  |
| *Pinus halepensis* | Pinaceae |  | 1 | 1 | √ | |  |
| *Quercus cerris* var. *cerris* | Fagaceae | 1 |  | 1 | √ | |  |
| *Quercus brantii* | Fagaceae | 1 |  | 1 | √ | |  |
| *Quercus pinnatifida* | Fagaceae |  | 1 | 1 |  | |  |
| Total | 13 | 18 | 34 | 52 | 14 | |  |

^a^ Basionym: *Quercus coccifera* ssp. *calliprinos* (Webb.) Holm

^b^ Synonymous with *Amygdalus orientalis* Mill.

^c^ Synonymous with *Amygdalus spartioides* Spach.

^†^ From private nurseries at the time of the survey
